# Pilot study to evaluate hypercoagulation and inflammation using rotational thromboelastometry and calprotectin in COVID-19 patients

**DOI:** 10.1371/journal.pone.0269738

**Published:** 2023-01-06

**Authors:** Sophia Stanford, Ashok Roy, Catherine Rea, Ben Harris, Antony Ashton, Sarah Mangles, Tamara Everington, Rayan Taher, Daniel Burns, Emily Arbuthnot, Tom Cecil

**Affiliations:** 1 Peritoneal Malignancy Institute, Hampshire Hospitals NHS Foundation Trust, Basingstoke, Hampshire, United Kingdom; 2 East Sussex Healthcare NHS Foundation Trust, Eastbourne, United Kingdom; 3 Anaesthetics and Critical Care, Hampshire Hospitals NHS Foundation Trust, Basingstoke, Hampshire, United Kingdom; 4 Haemophilia, Haemostasis and Thrombosis Centre, Hampshire Hospitals NHS Foundation Trust, Basingstoke, Hampshire, United Kingdom; Karolinska Institutet, SWEDEN

## Abstract

**Introduction:**

Abnormal coagulation and inflammation are hallmarks of SARs-COV-19. Stratifying affected patients on admission to hospital may help identify those who at are risk of developing severe disease early on. Rotational Thromboelastometry (ROTEM) is a point of care test that can be used to measure abnormal coagulation and calprotectin is a measure of inflammation.

**Aim:**

Assess if ROTEM can measure hypercoagulability on admission and identify those who will develop severe disease early on. Assess if calprotectin can measure inflammation and if there is a correlation with ROTEM and calprotectin.

**Methods:**

COVID-19 patients were recruited on admission and ROTEM testing was undertaken daily for a period of 7 days. Additionally inflammatory marker calprotectin was also tested for the same period.

**Results:**

33 patients were recruited to the study out of which 13 were admitted to ITU and 20 were treated on the ward. ROTEM detected a hypercoagulable state on admission but did not stratify between those admitted to a ward or escalated to ITU. Calprotectin levels were raised but there was no statistical difference (p = 0.73) between patients admitted to a ward or escalated to ITU. Significant correlations were observed between FIBA5 (r = 0.62; p<0.00), FIBCFT (r = -0.57; p<0.00), FIBMCF (r = 0.64; p<0.00) and INMCF (r = 0.57; p<0.00) and calprotectin.

**Conclusion:**

COVID-19 patients were hypercoagulable on admission. The correlations between ROTEM and calprotectin underline the interactions between inflammation and coagulation.

## Introduction

Patients infected with the coronavirus SARS-CoV-2 leading to the coronavirus disease 2019 (COVID-19) become symptomatic after an average incubation of 5.2 days [1]. Severely affected patients develop shortness of breath at a median of 8 days from illness onset, with acute respiratory distress syndrome, pneumonia developing at day 9 and admission to Intensive Therapy Unit (ITU) at day 10.5 [2].

Biologically, severe COVID-19 is characterised by the production of proinflammatory cytokines [3]. There is extensive cross talk between inflammation and coagulation systems in response to invasion by pathogens [4–6]. Reflecting this, several studies have reported abnormalities in laboratory markers of coagulation and fibrinolysis in COVID-19 patients [7–11]. Retrospective data indicates that there is significantly more derangement in coagulation parameters (namely prothrombin time (PT) and D-Dimer) at the point of admission in patients who don’t survive, versus those who do [12]. These changes in individual coagulation parameters point to a disruption in haemostasis, but do not provide any guidance as to the biological effect of the changes. Traditional coagulation tests provide a snapshot of a particular aspect of coagulation in cell depleted plasma, but do not provide an assessment of overall haemostasis. Rotational thromboelastometry (ROTEM^®^) provides a composite assessment of the dynamic process of clot initiation, thrombin generation and whole blood clot formation which is arguably more representative of physiological processes [13]. A number of studies have used ROTEM^®^ to assess haemostatic status and demonstrate a prothrombotic phenotype in patients admitted to ITU [14–16]. Furthermore, studies have also shown that ROTEM^®^ can be potentially used as a predictor for thrombosis and disease severity on admission [17–20]. Inflammatory responses such as enhanced neutrophil infiltration in tissue and organs of patients infected with COVID-19 has been observed [21]. Consequently, neutrophil’s role in COVID-19 as well as surrogate markers of neutrophilic activation such as calprotectin, have been investigated to understand disease progression and possible complications [22–26]. The potential of calprotectin as a prognostic marker and its influence on coagulation needed to be further explored.

In this study we investigated if COVID-19 patients demonstrated a prothrombotic phenotype as measured by ROTEM *Sigma* and if it could be used as a predictor of disease severity on admission to hospital. Calprotectin was also evaluated as a marker of inflammation and disease progression. Correlations between ROTEM and calprotectin was undertaken to understand its role in contributing to hypercoagulation in COVID-19 patients. To evaluate the optimal levels of ROTEM and calprotectin values which escalate admission of patients to ITU, a sample size of 30 was deemed adequate to show statistical significance.

## Materials and methods

In line with the Declaration of Helsinki, ethical approval was obtained from the Cornwall and Plymouth Research Ethics Committee (IRAS No: 284755). Written informed consent was obtained from patients. As part of the ethical approval where patients had severe disease and were not able to consent, assent was obtained from an independent medical practitioner who was not involved in the direct care of the patient. Patients who were admitted to hospital with a suspected/known diagnosis of COVID-19 (RT-PCR) and over the age of 18 were included in the study. Patients who had a negative COVID-19 test after recruitment were subsequently excluded from analysis. Any patients with a previous history of coagulation disorders including venous thromboembolisn within 6 months and those on anticoagulant therapy excluding low molecular weight heparin thromboprophylaxis were excluded from the study. Admitted patients either went to a normal ward or to ITU depending on clinical severity. Patients with mild to moderate respiratory failure were administered oxygen via nasal cannula and venturi mask upto a maximum of 15 L/minute, if this was insufficient continuous positive airway pressure (CPAP) was administered. Those with severe respiratory failure were treated in ITU with invasive ventilation support. On admission patients were treated with a single prophylactic dose of enoxaparin in the emergency department and then transferred to either a ward or ITU. Enoxaparin was administered twice daily (6am/6pm) as anticoagulation treatment and dose was decided on body weight (Enoxaparin 40mg if <100kg, 60mg if 100-150Kg and 80mg if >150kg). All patients received Enoxaparin and were also given a stat dose of vitamin K IV with the first dose of enoxaparin. This was given as testing at the start of the pandemic had shown several patients had low protein C or S on admission. For patients admitted to ITU the Intensive Care National Audit and Research Centre (ICNARC) score was calculated (Table 1, results section).

**Table 1:** Demographic data of enrolled patients admitted to hospital with COVID-19 (n = 33).

**Gender**	Female	14 (42.4%)
	Male	19 (57.8%)
**Age**	Median (IQR)	52 years (41.0–62.5)
**Smoking Status**	Yes	2 (6%)
	No	24(72%)
	Vape	2 (6%)
	Ex-smoker	5 (21.21%)
**Comorbidities**	Diabetes	9 (27.3%)
	Hypertension	8 (24.2%)
	Cardiovascular	6 (18.2%)
	Cerebrovascular	1 (3%)
	Asthma/COPD	6 (18.2%)
	Malignancy	1 (3%)
	Other diseases	15 (45.5%)
	Nil	6 (18.2%)
**COVID-19 diagnosed**	On admission	25 (75.8%)
	Later in ward	8 (24.2%)
**Admission Unit**	Ward	20 (60.6%)
	ITU	13 (39.4%)
**ICNARC score (ITU admissions)**	Median (IQR)	9.5 (12–21.5)
**Length of Stay**	Median (IQR)	8 days (5–16)
**Outcome / Event**	VTE	1 (3.0%)
	Discharge home	29 (88.0%)
	Deceased	4 (12.0%)

Blood was taken on admission and everyday for the first 7 days of hospital admission patients discharged before 7 days were also included in the study. The follow-up blood were taken approximately 3 hours after anticoagulant treatment. Rotational thromboelastometry was tested within 4 hours of taking the blood and the plasma aliquots were frozen for batch testing of calprotectin.

### Rotational thromboelastometry

ROTEM *Sigma* (Werfen UK) is a viscoelastic point of care test with a fully automated system containing a sample handler and cartridge that uses whole blood to measure coagulation [27]. All tests were analysed for 60 minutes and within 4 hours of sample collection. Reference ranges were obtained from healthy volunteer data (n = 26) previously collected.

Data from three ROTEM channels are presented, FIBTEM, INTEM and EXTEM. FIBTEM uses cytochalasin D to inhibit platelet activity and provide a clot tracing that reflects the presence of fibrinogen. It provides information on fibrin formation and polymerisation without platelet contribution [28]. In EXTEM tissue factor is used to initiate the extrinsic clotting cascade. The INTEM test uses ellagic acid to initiate clotting via the intrinsic pathway. The EXTEM and INTEM tests provide information on the extrinsic and intrinsic pathways respectively. The viscoelastic characteristics of the clot are measured from clot formation to lysis and include clotting time (CT), clot formation time (CFT), maximum clot firmess (MCF), A5-A30 (measure of clot firmess from 5–30 minutes) and maximum lysis (ML) which is the degree of fibrinolysis relative to MCF achieved during measurement and is reported as percentage of clot firmness lost [29]. A shortened CFT with an increased MCF is indicative of a hypercoagulable state.

### Calprotectin

Circulating calprotectin levels were measured in plasma using a chemiluminescent immunoassay (QUANTA Flash, Werfen, UK) according to manufacturer’s instructions. The chemiluminescent immunoassay utilises a predefined lot specific master curve that is stored in the reagent cartridge barcode.

### Statistics

Demographics of the patient population are presented as frequencies and percentages. All continuous variables are presented with medians and interquartile ranges (IQR). Mann-Whitney U test was used to test whether there were significant differences between patients admitted to ITU vs ward for ROTEM and calprotectin. We included individuals in the ITU cohort if they were admitted to the high dependency unit (HDU) or intensive treatment unit (ITU) at any point during admission (n = 13). The ward cohort represents the remaining individuals in the study (n = 20). Spearman’s rank correlation coefficients were calculated to assess associations between variables. A Bonferroni correction was used to evaluate significance with both tests. A p-value below 0.05 at a 95% confidence interval was considered significant. Analysis was performed in Anaconda 3 with Python 3.8.8.

## Results

Blood was obtained from 47 patients admitted to Hampshire Hospitals from October 2020-April 2021. 14 patients were excluded as they tested negative for COVID-19, 33 who tested positive were included in the study. Rotational thromboelastometry demonstrated a hypercoagulable state compared to previously collected data from healthy individuals, demographic data are presented in Table 1. On admission significant differences between COVID-19 patients and healthy individuals were observed for FIBTEM CT (p<0.001) and MCF (p<0.001), EXTEM CT, CFT, MCF and ML (p<0.002) and INTEM CT (p<0.028), MCF (p<0.004) and ML (p<0.001).

### ITU vs ward

Comparison was made between ITU and wards patients, median values on admission were compared between the two groups. No Significant differences were seen between those admitted to ITU versus those admitted to the ward for any of the ROTEM parameters, Table 2. Similarly, no significant differences were observed in calprotectin between the two groups. Longitudinal data for all ROTEM tests at the various time points for both ITU and ward cohorts are presented in Appendix 1.

**Table 2:** ROTEM parameters between those admitted to ward and ITU. The median values on admission are presented in the table.

ROTEM parameters (normal ranges)	Ward (n = 20) Median (IQR)	ITU (n = 13) Median (IQR)	p value
FIBCT (46–84 s)	70 (65–77)	67 (66–74)	0.34
FIBMCF (6–21 mm)	28 (25–33)	26 (24–30)	0.15
EXCT (52–83 s)	70 (60–73)	67 (59–71)	0.31
EXCFT (46–149 s)	46 (43–57)	57 (54–61)	0.97
EXMCF (55–72 mm)	71 (68–74)	72 (69–73)	0.62
EXML (0–15%)	5 (3–10)	4 (2–6)	0.06
INCT (168–212 s)	184 (162–197)	185 (176–194)	0.50
INCFT (62–130 s)	55 (49–71)	58 (55–70)	0.83
INMCF (51–69 mm)	68 (66–70)	67 (66–70)	0.51
INML (0–15%)	5 (2–8)	4 (3–6)	0.23

**Fig 1:**
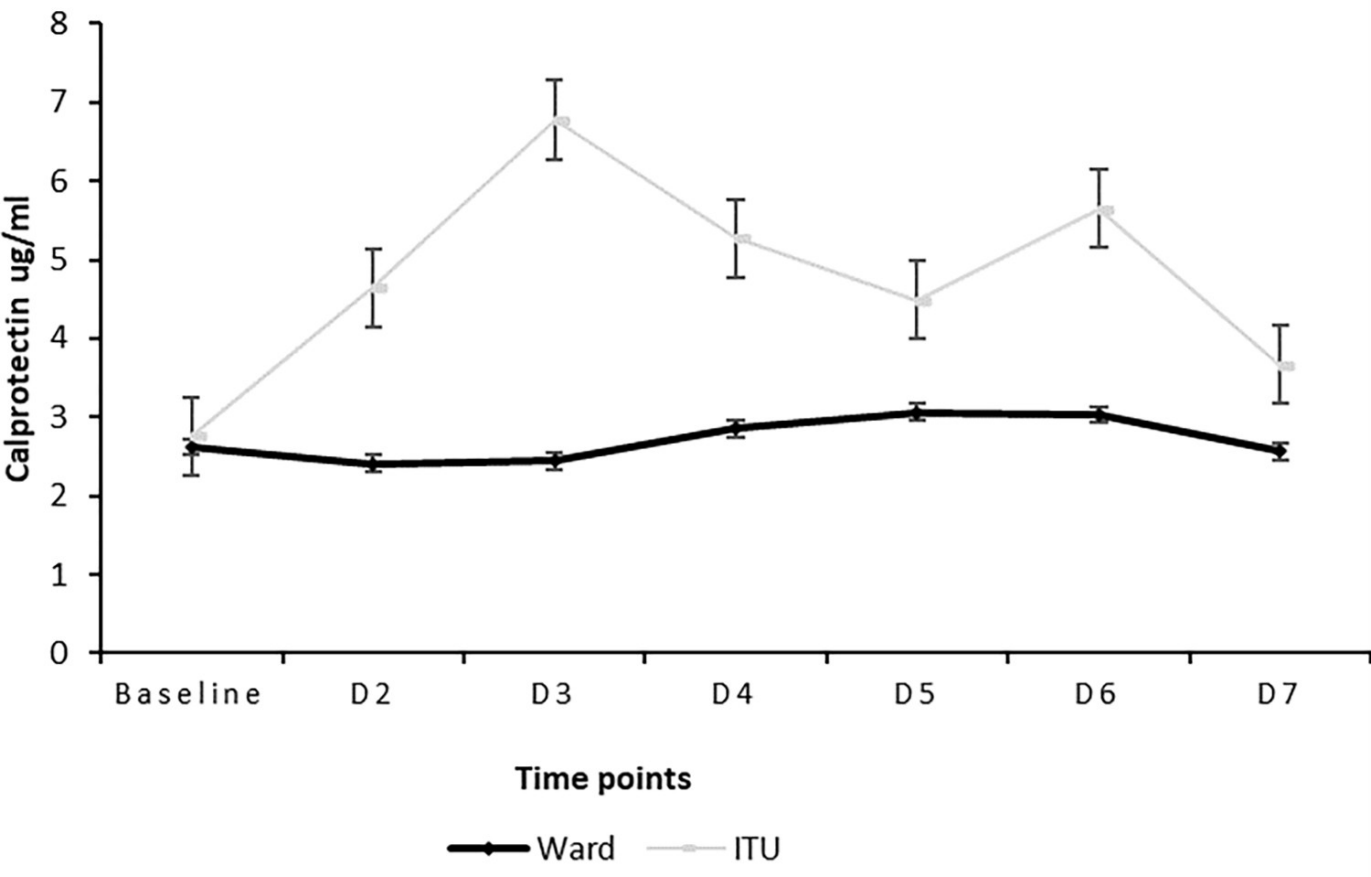
Calprotectin trend between ITU patients and those on the ward for admission through to week 2. Normal range for calprotectin is <1.99μg/ml.

Although not statistically significant (p = 0.73) the calprotectin values were higher in the ITU patients; [median: 4.17; IQR:2.17–8.52] compared to those on the ward; [median:2.08; IQR: 1.11–3.53] the trends are in Fig 1. Averages for each time point are presented. Significant correlations were observed between FIBTEM, EXTEM and INTEM parameters and calprotectin, Table 3.

**Table 3:** Spearman rank correlations between ROTEM and coagulation and calprotectin.

ROTEM parameters	Calprotectin r (p value)
FIBA5	0.62 (0.00)***
FIBCFT	-0.57 (0.00)***
FIBMCF	0.64 (0.00)***
EXA5	0.43 (0.01)**
EXMCF	0.46 (0.01)**
INCT	0.07 (0.68)
INA5	0.41 (0.02)*
INCFT	-0.41 (0.01)**
INMCF	0.57 (0.00)***
INLI30	0.1 (0.56)

* p-value less than 0.05.

** p-value less than 0.01.

*** p-value less than 0.001 (significant after Bonferroni correction).

## Discussion

This pilot study confirmed hypercoagulability in COVID-19 patients on admission based on ROTEM parameters. However, ROTEM was not able to stratify on admission those who were escalated to ITU and those who were not.

This is in contrast with other studies that have also assessed if ROTEM can be used as a predictor of disease severity on admission. Almskog et al undertook ROTEM testing in COVID-19 patients who were admitted to regular wards and specialised ventilation support. All hospitalised patients with COVID-19 patients showed elevated values of EXMCF and FIBMCF on admission to hospital and this hypercoagulable pattern was more prominent in the severely ill patients compared to those on regular wards [17]. Similarly, Boscolo et al investigated the difference in MCF values between those patients admitted to internal medicine wards and ICU. They found that ICU patients had a significantly higher FIBMCF compared to those admitted to the internal medicine wards [19]. Lastly, ROTEM parameters were also measured in COVID-19 patients with varying severities of pneumonia; a hypercoagulable ROTEM pattern due to shortened EXCT, higher than normal EXMCF and FIBMCF and shortened EXCFT, high clot strength and hypofibrinolysis in advanced disease and patients with high levels of IL-6 were observed [20]. It is likely that we did not see a difference between the two groups because of the small numbers of patients in our ITU cohort. Other confounders such as age, gender, disease severity, COVID-19 variants, race, ethnicity and smoking status may also have impacted the results of this study. It has been shown that patients aged 70 and over were more often observed to have confirmed COVID-19 infection, severe disease and at a higher risk of ITU admission and death [30]. The median age of our patient cohort was 52 years and therefore may have had less severe disease. COVID-19 affects men more severely than women, in our study, there were marginally more men than women but not enough to assess disease severity based on gender. At the time of this study there was no knowledge of the COVID-19 variants which may also have had an impact on the results. Coagulation status is affected by race and ethnicity, as this study consisted mainly of Caucasian patients this may have impacted the results [31]. Smoking induces a state of hypercoagulability and influences platelets and haemostatic mechanisms [32], in this cohort of patients there were more non-smokers than smokers which may have impacted the level of hypercoagulation observed.

Calprotectin levels have been shown to be elevated in COVID-19 patients [22–24]. In our study we found that calprotectin levels were raised overall, and patients admitted to ITU although not statistically significant had higher calprotectin levels compared to those admitted to a ward. Various studies have reported raised calprotectin levels in COVID-19 patients. A marked difference in the serum calprotectin levels between COVID-19 survivors and non-survivors has been observed [22]. Another study demonstrated that reduced frequency of non-classical monocytes along with raised serum calprotectin levels has the potential to identify patients who will develop severe COVID-19 [25]. A review of calprotectin in COVID-19 provided evidence suggesting that calprotectin could be useful in assessing disease severity [33]. Similar to our ROTEM results, it is likely we did not see a statistical difference between the two groups because of the small numbers in the ITU cohort. Moreover, it is likely that the younger median age of our cohort with potentially less severe disease may have impacted our results. Plasma calprotectin has been found to be independently associated with smoking status with current smokers having higher levels of calprotectin compared to non-smokers- in type 2 diabetes [34]. This could also be the case in COVID-19 patients as in this study there were more non-smokers than smokers which could have impacted the levels of circulating calprotectin.

We are the first to report significant correlations between FIBA5, FIBCFT and FIBMCF and calprotectin. It is known that fibrinogen is an acute phase reactant which is increased during an inflammatory response [35]. In COVID-19 patients increased fibrinogen levels have been associated with excessive inflammation [36]. We suggest that the significant correlation we have observed between fibrinogen and calprotectin is because of fibrinogen’s role in inflammation. Calprotectin also correlated with INMCF which measures the intrinsic pathway of coagulation. FVIII and VWF which are part of the intrinsic pathway of coagulation are also linked to inflammation [37–39] and are known to be raised in COVID-19 patients [39–43]. Similar to fibrinogen, the link that FVIII and vWF have to inflammation could be the reason for the significant correlation calprotectin and INMCF. These interesting findings further highlights the role of inflammation in COVID-19 and its impact on coagulation which is measurable using ROTEM and should be investigated further. In addition to undertaking ROTEM testing in COVID-19 patients, consideration should be given to measuring calprotectin levels. ROTEM is a point of care test with quick turnaround times that gives an overview of coagulation. Calprotectin has the potential to be used as a marker of inflammation in these patients.

In summary, ROTEM detected a hypercoagulable state in COVID-19 patients but could not stratify severity of disease on admission in this pilot study. Significant correlations were observed between ROTEM and calprotectin demonstrating the synergy that exists between inflammation and coagulation. Whilst interpreting coagulation and inflammatory responses in COVID-19 it is also important to take into consideration other confounding factors that affect coagulation and inflammation.

### Limitations

No definite conclusions can be drawn from this pilot study because of the small sample size and confounding factors. The study consisted mainly of Caucasian patients and as coagulation status and predisposition to the development of coagulopathies varies between race and ethnicity [31] this limits the generalisability of our study.

## Supporting information

S1 Appendix Comparison of ITU vs ward for the various tests over time. **Fig 1:** Median (IQR) values of FIBCT for COVID-19 patients over the first 7 days of admission to hospital. Comparison of FIBCT values between ITU and ward admissions. **Fig 2:** Median (IQR) values of FIBMCF for COVID-19 patients over the first 7 days of admission to hospital. Comparison of FIBMCF values between ITU and ward admissions. **Fig 3:** Median (IQR) values of EXCT for COVID-19 patients over the first 7 days of hospital admission. Comparison of EXCT values between ITU and ward admissions. **Fig 4:** Median (IQR) values of EXCFT for COVID-19 patients over the first 7 days of hospital admission. Comparison of EXCFT values between ITU and ward admissions. **Fig 5:** Median (IQR) values of EXMCF for COVID-19 patients over the first 7 days of hospital admission. Comparison of EXMCF values between ITU and ward patients. **Fig 6:** Median (IQR) values for EXML for COVID-19 patients over the first 7 days of hospital admission. Comparison of EXML values between ITU and ward patients. **Fig 7:** Median (IQR) values for INCT for COVID-19 patients over the first 7 days of hospital admission. Comparison of INCT values between ITU and ward patients. **Fig 8:** Median (IQR) values for INCFT for COVID-19 patients over the first 7 days of hospital admission. Comparison of INCFT values between ITU and ward patients. **Fig 9:** Median (IQR) values for INMCF for COVID-19 patients over the first 7 days of hospital admission. Comparison of INMCF values between the ITU and ward patients. **Fig 10:** Median (IQR) values for INML for COVID-19 patients over the first 7 days of hospital admission. Comparison of INML values between ITU and ward patients.
(ZIP)
