# Supplementary figures and images for: Pilot study to evaluate hypercoagulation and inflammation using rotational thromboelastometry and calprotectin in COVID-19 patients
Source: PLoS One. 2023 Jan 6;18(1):e0269738. doi: 10.1371/journal.pone.0269738 (PMC9821718; doi:10.1371/journal.pone.0269738)

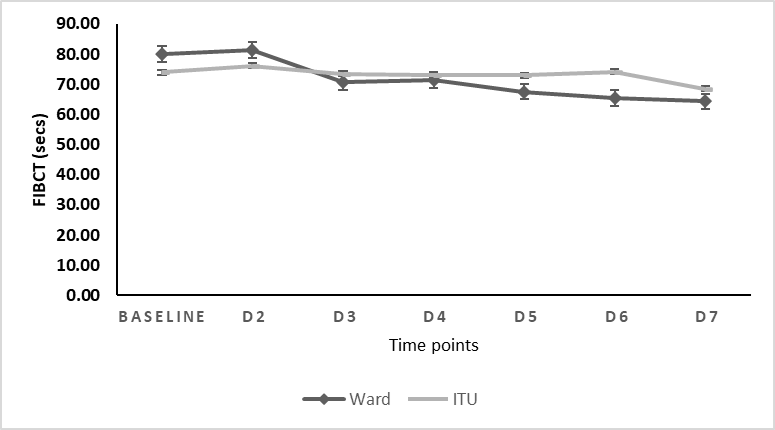

Supplement: S1 Appendix — Fig 1: Median (IQR) values of FIBCT for COVID-19 patients over the first 7 days of admission to hospital. Comparison of FIBCT values between ITU and ward admissions. Fig 2: Median (IQR) values of FIBMCF for COVID-19 patients over the first 7 days of admission to hospital. Comparison of FIBMCF values between ITU and ward admissions. Fig 3: Median (IQR) values of EXCT for COVID-19 patients over the first 7 days of hospital admission. Comparison of EXCT values between ITU and ward admissions. Fig 4: Median (IQR) values of EXCFT for COVID-19 patients over the first 7 days of hospital admission. Comparison of EXCFT values between ITU and ward admissions. Fig 5: Median (IQR) values of EXMCF for COVID-19 patients over the first 7 days of hospital admission. Comparison of EXMCF values between ITU and ward patients. Fig 6: Median (IQR) values for EXML for COVID-19 patients over the first 7 days of hospital admission. Comparison of EXML values between ITU and ward patients. Fig 7: Median (IQR) values for INCT for COVID-19 patients over the first 7 days of hospital admission. Comparison of INCT values between ITU and ward patients. Fig 8: Median (IQR) values for INCFT for COVID-19 patients over the first 7 days of hospital admission. Comparison of INCFT values between ITU and ward patients. Fig 9: Median (IQR) values for INMCF for COVID-19 patients over the first 7 days of hospital admission. Comparison of INMCF values between the ITU and ward patients. Fig 10: Median (IQR) values for INML for COVID-19 patients over the first 7 days of hospital admission. Comparison of INML values between ITU and ward patients. (ZIP) [file pone.0269738.s001.zip › Appendix Figure 1.tif]

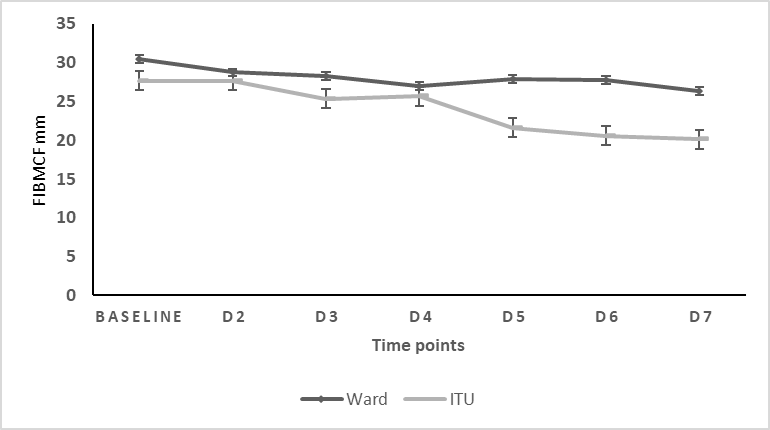

Supplement: S1 Appendix — Fig 1: Median (IQR) values of FIBCT for COVID-19 patients over the first 7 days of admission to hospital. Comparison of FIBCT values between ITU and ward admissions. Fig 2: Median (IQR) values of FIBMCF for COVID-19 patients over the first 7 days of admission to hospital. Comparison of FIBMCF values between ITU and ward admissions. Fig 3: Median (IQR) values of EXCT for COVID-19 patients over the first 7 days of hospital admission. Comparison of EXCT values between ITU and ward admissions. Fig 4: Median (IQR) values of EXCFT for COVID-19 patients over the first 7 days of hospital admission. Comparison of EXCFT values between ITU and ward admissions. Fig 5: Median (IQR) values of EXMCF for COVID-19 patients over the first 7 days of hospital admission. Comparison of EXMCF values between ITU and ward patients. Fig 6: Median (IQR) values for EXML for COVID-19 patients over the first 7 days of hospital admission. Comparison of EXML values between ITU and ward patients. Fig 7: Median (IQR) values for INCT for COVID-19 patients over the first 7 days of hospital admission. Comparison of INCT values between ITU and ward patients. Fig 8: Median (IQR) values for INCFT for COVID-19 patients over the first 7 days of hospital admission. Comparison of INCFT values between ITU and ward patients. Fig 9: Median (IQR) values for INMCF for COVID-19 patients over the first 7 days of hospital admission. Comparison of INMCF values between the ITU and ward patients. Fig 10: Median (IQR) values for INML for COVID-19 patients over the first 7 days of hospital admission. Comparison of INML values between ITU and ward patients. (ZIP) [file pone.0269738.s001.zip › Appendix Figure 2.tif]

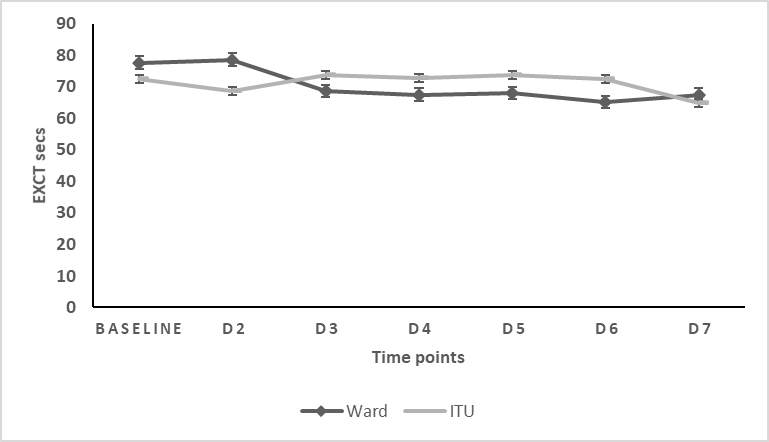

Supplement: S1 Appendix — Fig 1: Median (IQR) values of FIBCT for COVID-19 patients over the first 7 days of admission to hospital. Comparison of FIBCT values between ITU and ward admissions. Fig 2: Median (IQR) values of FIBMCF for COVID-19 patients over the first 7 days of admission to hospital. Comparison of FIBMCF values between ITU and ward admissions. Fig 3: Median (IQR) values of EXCT for COVID-19 patients over the first 7 days of hospital admission. Comparison of EXCT values between ITU and ward admissions. Fig 4: Median (IQR) values of EXCFT for COVID-19 patients over the first 7 days of hospital admission. Comparison of EXCFT values between ITU and ward admissions. Fig 5: Median (IQR) values of EXMCF for COVID-19 patients over the first 7 days of hospital admission. Comparison of EXMCF values between ITU and ward patients. Fig 6: Median (IQR) values for EXML for COVID-19 patients over the first 7 days of hospital admission. Comparison of EXML values between ITU and ward patients. Fig 7: Median (IQR) values for INCT for COVID-19 patients over the first 7 days of hospital admission. Comparison of INCT values between ITU and ward patients. Fig 8: Median (IQR) values for INCFT for COVID-19 patients over the first 7 days of hospital admission. Comparison of INCFT values between ITU and ward patients. Fig 9: Median (IQR) values for INMCF for COVID-19 patients over the first 7 days of hospital admission. Comparison of INMCF values between the ITU and ward patients. Fig 10: Median (IQR) values for INML for COVID-19 patients over the first 7 days of hospital admission. Comparison of INML values between ITU and ward patients. (ZIP) [file pone.0269738.s001.zip › Appendix Figure 3.tif]

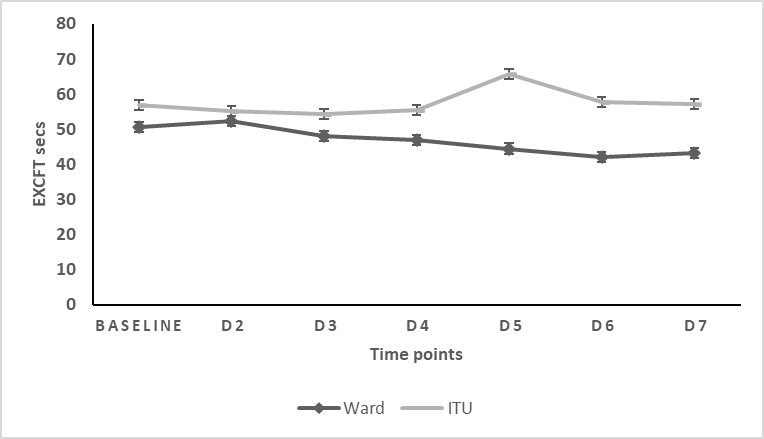

Supplement: S1 Appendix — Fig 1: Median (IQR) values of FIBCT for COVID-19 patients over the first 7 days of admission to hospital. Comparison of FIBCT values between ITU and ward admissions. Fig 2: Median (IQR) values of FIBMCF for COVID-19 patients over the first 7 days of admission to hospital. Comparison of FIBMCF values between ITU and ward admissions. Fig 3: Median (IQR) values of EXCT for COVID-19 patients over the first 7 days of hospital admission. Comparison of EXCT values between ITU and ward admissions. Fig 4: Median (IQR) values of EXCFT for COVID-19 patients over the first 7 days of hospital admission. Comparison of EXCFT values between ITU and ward admissions. Fig 5: Median (IQR) values of EXMCF for COVID-19 patients over the first 7 days of hospital admission. Comparison of EXMCF values between ITU and ward patients. Fig 6: Median (IQR) values for EXML for COVID-19 patients over the first 7 days of hospital admission. Comparison of EXML values between ITU and ward patients. Fig 7: Median (IQR) values for INCT for COVID-19 patients over the first 7 days of hospital admission. Comparison of INCT values between ITU and ward patients. Fig 8: Median (IQR) values for INCFT for COVID-19 patients over the first 7 days of hospital admission. Comparison of INCFT values between ITU and ward patients. Fig 9: Median (IQR) values for INMCF for COVID-19 patients over the first 7 days of hospital admission. Comparison of INMCF values between the ITU and ward patients. Fig 10: Median (IQR) values for INML for COVID-19 patients over the first 7 days of hospital admission. Comparison of INML values between ITU and ward patients. (ZIP) [file pone.0269738.s001.zip › Appendix Figure 4.tif]

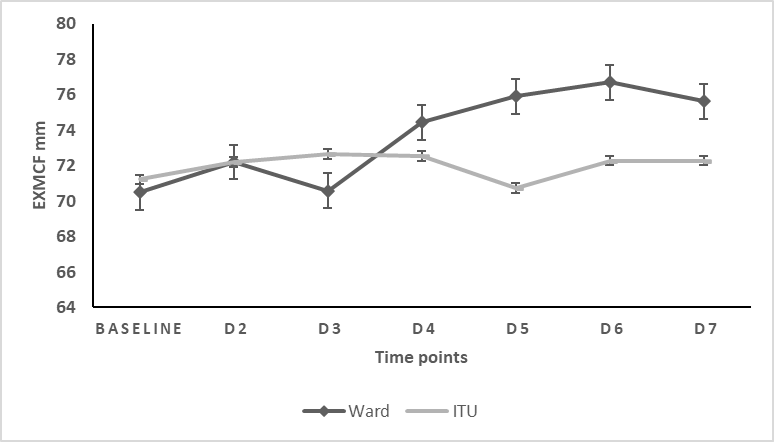

Supplement: S1 Appendix — Fig 1: Median (IQR) values of FIBCT for COVID-19 patients over the first 7 days of admission to hospital. Comparison of FIBCT values between ITU and ward admissions. Fig 2: Median (IQR) values of FIBMCF for COVID-19 patients over the first 7 days of admission to hospital. Comparison of FIBMCF values between ITU and ward admissions. Fig 3: Median (IQR) values of EXCT for COVID-19 patients over the first 7 days of hospital admission. Comparison of EXCT values between ITU and ward admissions. Fig 4: Median (IQR) values of EXCFT for COVID-19 patients over the first 7 days of hospital admission. Comparison of EXCFT values between ITU and ward admissions. Fig 5: Median (IQR) values of EXMCF for COVID-19 patients over the first 7 days of hospital admission. Comparison of EXMCF values between ITU and ward patients. Fig 6: Median (IQR) values for EXML for COVID-19 patients over the first 7 days of hospital admission. Comparison of EXML values between ITU and ward patients. Fig 7: Median (IQR) values for INCT for COVID-19 patients over the first 7 days of hospital admission. Comparison of INCT values between ITU and ward patients. Fig 8: Median (IQR) values for INCFT for COVID-19 patients over the first 7 days of hospital admission. Comparison of INCFT values between ITU and ward patients. Fig 9: Median (IQR) values for INMCF for COVID-19 patients over the first 7 days of hospital admission. Comparison of INMCF values between the ITU and ward patients. Fig 10: Median (IQR) values for INML for COVID-19 patients over the first 7 days of hospital admission. Comparison of INML values between ITU and ward patients. (ZIP) [file pone.0269738.s001.zip › Appendix figure 5.tif]

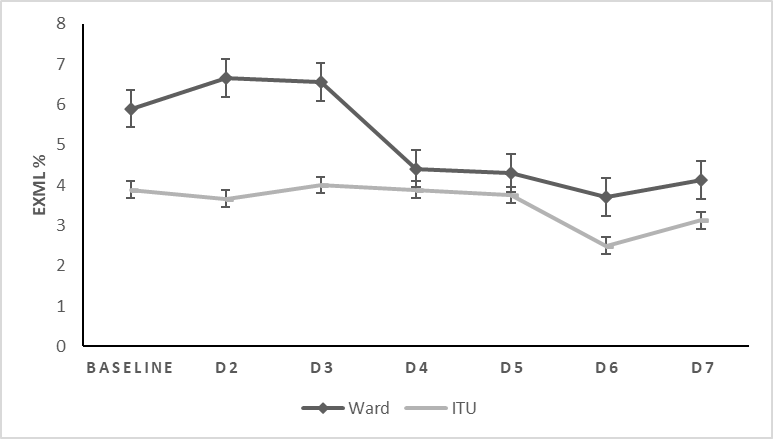

Supplement: S1 Appendix — Fig 1: Median (IQR) values of FIBCT for COVID-19 patients over the first 7 days of admission to hospital. Comparison of FIBCT values between ITU and ward admissions. Fig 2: Median (IQR) values of FIBMCF for COVID-19 patients over the first 7 days of admission to hospital. Comparison of FIBMCF values between ITU and ward admissions. Fig 3: Median (IQR) values of EXCT for COVID-19 patients over the first 7 days of hospital admission. Comparison of EXCT values between ITU and ward admissions. Fig 4: Median (IQR) values of EXCFT for COVID-19 patients over the first 7 days of hospital admission. Comparison of EXCFT values between ITU and ward admissions. Fig 5: Median (IQR) values of EXMCF for COVID-19 patients over the first 7 days of hospital admission. Comparison of EXMCF values between ITU and ward patients. Fig 6: Median (IQR) values for EXML for COVID-19 patients over the first 7 days of hospital admission. Comparison of EXML values between ITU and ward patients. Fig 7: Median (IQR) values for INCT for COVID-19 patients over the first 7 days of hospital admission. Comparison of INCT values between ITU and ward patients. Fig 8: Median (IQR) values for INCFT for COVID-19 patients over the first 7 days of hospital admission. Comparison of INCFT values between ITU and ward patients. Fig 9: Median (IQR) values for INMCF for COVID-19 patients over the first 7 days of hospital admission. Comparison of INMCF values between the ITU and ward patients. Fig 10: Median (IQR) values for INML for COVID-19 patients over the first 7 days of hospital admission. Comparison of INML values between ITU and ward patients. (ZIP) [file pone.0269738.s001.zip › Appendix figure 6.tif]

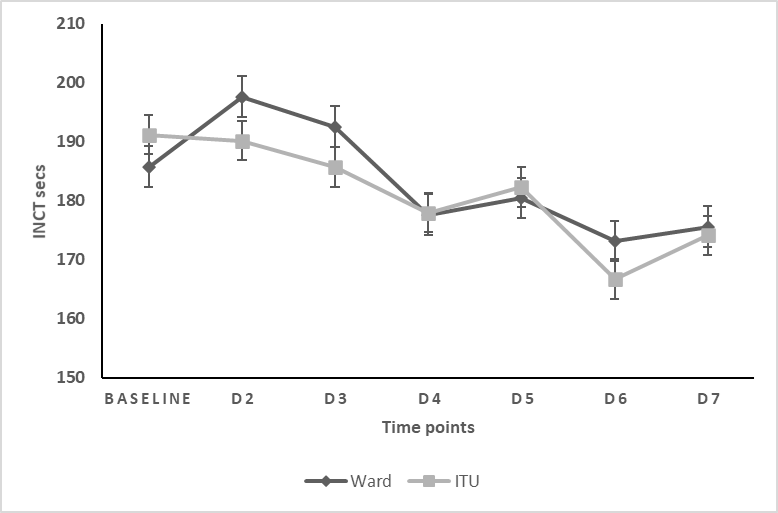

Supplement: S1 Appendix — Fig 1: Median (IQR) values of FIBCT for COVID-19 patients over the first 7 days of admission to hospital. Comparison of FIBCT values between ITU and ward admissions. Fig 2: Median (IQR) values of FIBMCF for COVID-19 patients over the first 7 days of admission to hospital. Comparison of FIBMCF values between ITU and ward admissions. Fig 3: Median (IQR) values of EXCT for COVID-19 patients over the first 7 days of hospital admission. Comparison of EXCT values between ITU and ward admissions. Fig 4: Median (IQR) values of EXCFT for COVID-19 patients over the first 7 days of hospital admission. Comparison of EXCFT values between ITU and ward admissions. Fig 5: Median (IQR) values of EXMCF for COVID-19 patients over the first 7 days of hospital admission. Comparison of EXMCF values between ITU and ward patients. Fig 6: Median (IQR) values for EXML for COVID-19 patients over the first 7 days of hospital admission. Comparison of EXML values between ITU and ward patients. Fig 7: Median (IQR) values for INCT for COVID-19 patients over the first 7 days of hospital admission. Comparison of INCT values between ITU and ward patients. Fig 8: Median (IQR) values for INCFT for COVID-19 patients over the first 7 days of hospital admission. Comparison of INCFT values between ITU and ward patients. Fig 9: Median (IQR) values for INMCF for COVID-19 patients over the first 7 days of hospital admission. Comparison of INMCF values between the ITU and ward patients. Fig 10: Median (IQR) values for INML for COVID-19 patients over the first 7 days of hospital admission. Comparison of INML values between ITU and ward patients. (ZIP) [file pone.0269738.s001.zip › Appendix Figure 7.tif]

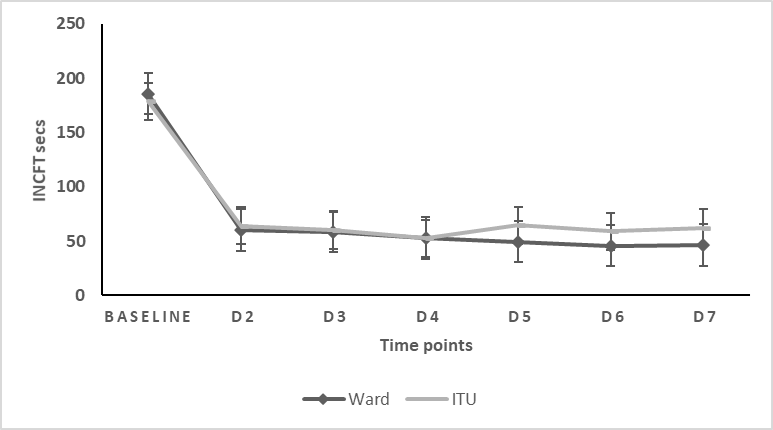

Supplement: S1 Appendix — Fig 1: Median (IQR) values of FIBCT for COVID-19 patients over the first 7 days of admission to hospital. Comparison of FIBCT values between ITU and ward admissions. Fig 2: Median (IQR) values of FIBMCF for COVID-19 patients over the first 7 days of admission to hospital. Comparison of FIBMCF values between ITU and ward admissions. Fig 3: Median (IQR) values of EXCT for COVID-19 patients over the first 7 days of hospital admission. Comparison of EXCT values between ITU and ward admissions. Fig 4: Median (IQR) values of EXCFT for COVID-19 patients over the first 7 days of hospital admission. Comparison of EXCFT values between ITU and ward admissions. Fig 5: Median (IQR) values of EXMCF for COVID-19 patients over the first 7 days of hospital admission. Comparison of EXMCF values between ITU and ward patients. Fig 6: Median (IQR) values for EXML for COVID-19 patients over the first 7 days of hospital admission. Comparison of EXML values between ITU and ward patients. Fig 7: Median (IQR) values for INCT for COVID-19 patients over the first 7 days of hospital admission. Comparison of INCT values between ITU and ward patients. Fig 8: Median (IQR) values for INCFT for COVID-19 patients over the first 7 days of hospital admission. Comparison of INCFT values between ITU and ward patients. Fig 9: Median (IQR) values for INMCF for COVID-19 patients over the first 7 days of hospital admission. Comparison of INMCF values between the ITU and ward patients. Fig 10: Median (IQR) values for INML for COVID-19 patients over the first 7 days of hospital admission. Comparison of INML values between ITU and ward patients. (ZIP) [file pone.0269738.s001.zip › Appendix figure 8.tif]

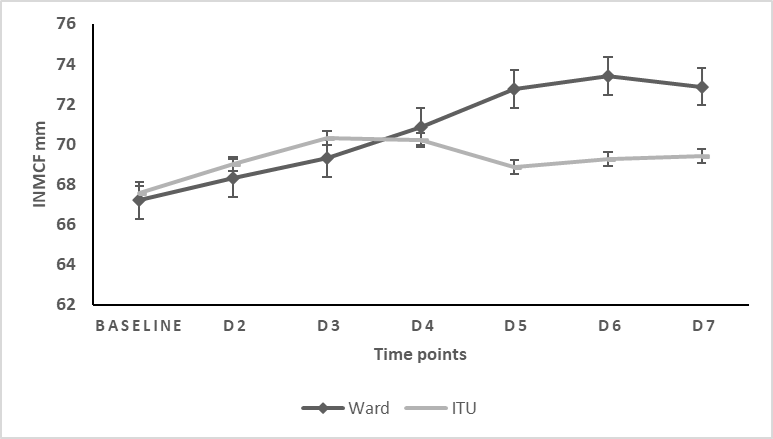

Supplement: S1 Appendix — Fig 1: Median (IQR) values of FIBCT for COVID-19 patients over the first 7 days of admission to hospital. Comparison of FIBCT values between ITU and ward admissions. Fig 2: Median (IQR) values of FIBMCF for COVID-19 patients over the first 7 days of admission to hospital. Comparison of FIBMCF values between ITU and ward admissions. Fig 3: Median (IQR) values of EXCT for COVID-19 patients over the first 7 days of hospital admission. Comparison of EXCT values between ITU and ward admissions. Fig 4: Median (IQR) values of EXCFT for COVID-19 patients over the first 7 days of hospital admission. Comparison of EXCFT values between ITU and ward admissions. Fig 5: Median (IQR) values of EXMCF for COVID-19 patients over the first 7 days of hospital admission. Comparison of EXMCF values between ITU and ward patients. Fig 6: Median (IQR) values for EXML for COVID-19 patients over the first 7 days of hospital admission. Comparison of EXML values between ITU and ward patients. Fig 7: Median (IQR) values for INCT for COVID-19 patients over the first 7 days of hospital admission. Comparison of INCT values between ITU and ward patients. Fig 8: Median (IQR) values for INCFT for COVID-19 patients over the first 7 days of hospital admission. Comparison of INCFT values between ITU and ward patients. Fig 9: Median (IQR) values for INMCF for COVID-19 patients over the first 7 days of hospital admission. Comparison of INMCF values between the ITU and ward patients. Fig 10: Median (IQR) values for INML for COVID-19 patients over the first 7 days of hospital admission. Comparison of INML values between ITU and ward patients. (ZIP) [file pone.0269738.s001.zip › Appendix Figure 9.tif]

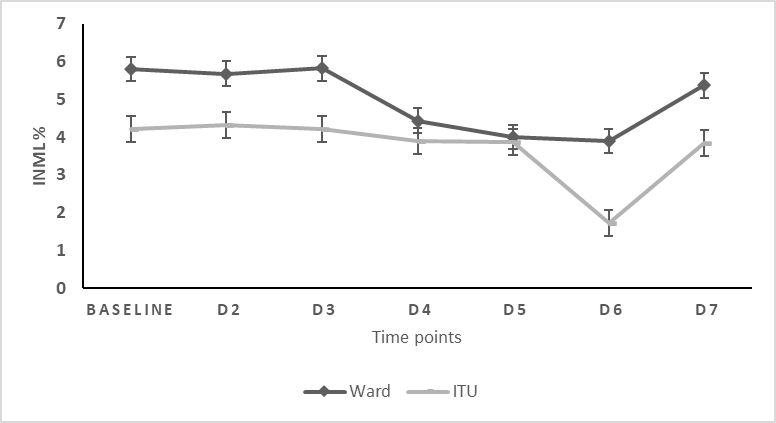

Supplement: S1 Appendix — Fig 1: Median (IQR) values of FIBCT for COVID-19 patients over the first 7 days of admission to hospital. Comparison of FIBCT values between ITU and ward admissions. Fig 2: Median (IQR) values of FIBMCF for COVID-19 patients over the first 7 days of admission to hospital. Comparison of FIBMCF values between ITU and ward admissions. Fig 3: Median (IQR) values of EXCT for COVID-19 patients over the first 7 days of hospital admission. Comparison of EXCT values between ITU and ward admissions. Fig 4: Median (IQR) values of EXCFT for COVID-19 patients over the first 7 days of hospital admission. Comparison of EXCFT values between ITU and ward admissions. Fig 5: Median (IQR) values of EXMCF for COVID-19 patients over the first 7 days of hospital admission. Comparison of EXMCF values between ITU and ward patients. Fig 6: Median (IQR) values for EXML for COVID-19 patients over the first 7 days of hospital admission. Comparison of EXML values between ITU and ward patients. Fig 7: Median (IQR) values for INCT for COVID-19 patients over the first 7 days of hospital admission. Comparison of INCT values between ITU and ward patients. Fig 8: Median (IQR) values for INCFT for COVID-19 patients over the first 7 days of hospital admission. Comparison of INCFT values between ITU and ward patients. Fig 9: Median (IQR) values for INMCF for COVID-19 patients over the first 7 days of hospital admission. Comparison of INMCF values between the ITU and ward patients. Fig 10: Median (IQR) values for INML for COVID-19 patients over the first 7 days of hospital admission. Comparison of INML values between ITU and ward patients. (ZIP) [file pone.0269738.s001.zip › Appendix Figure 10.tif]
